# Unsaturated polyurethanes degradable by conjugate substitution reactions with amines and carboxylate anions†

**DOI:** 10.1039/d3ra03461e

**Published:** 2023-07-07

**Authors:** Takumi Noda, Anri Tanaka, Yosuke Akae, Yasuhiro Kohsaka

**Affiliations:** a Faculty of Textile Science and Technology, Shinshu University 3-15-1 Tokida Ueda Nagano 386-8657 Japan; b Japan Society for the Promotion of Science (JSPS) Tokyo Japan; c Research Initiative for Supra-Materials (RISM), Shinshu University Japan kohsaka@shinshu-u.ac.jp +81-268-21-5488

## Abstract

Main-chain scission of polymers induces a significant decrease in molecular weight and accompanying changes in physical properties and is important for applications in materials engineering, such as in photoresists and adhesive dismantling. In this study, we focused on methacrylates substituted with carbamate groups at the allylic positions for the purpose of developing a mechanism that efficiently cleaves the main chain in response to chemical stimuli. Dimethacrylates substituted with hydroxy groups at the allylic positions were synthesized by the Morita–Baylis–Hillman reaction of diacrylates and aldehydes. The polyaddition with diisocyanates afforded a series of poly(conjugated ester-urethane)s. These polymers underwent a conjugate substitution reaction with diethylamine or acetate anion at 25 °C, resulting in main-chain scission accompanied by decarboxylation. A side reaction by the re-attack of the liberated amine end to the methacrylate skeleton proceeded, whereas it was suppressed for the polymers with an allylic substitute of the phenyl group. Therefore, the methacrylate skeleton substituted with phenyl and carbamate groups at the allylic position is an excellent decomposition point that induces selective and quantitative main-chain scission with weak nucleophiles, such as carboxylate anions.

## Abstract 2

Methacrylate skeletons substituted with phenyl and carbamate groups at the allylic position are excellent decomposition points that induce selective and quantitative main chain scission with weak nucleophiles such as acetate anions.

## Graphical abstract

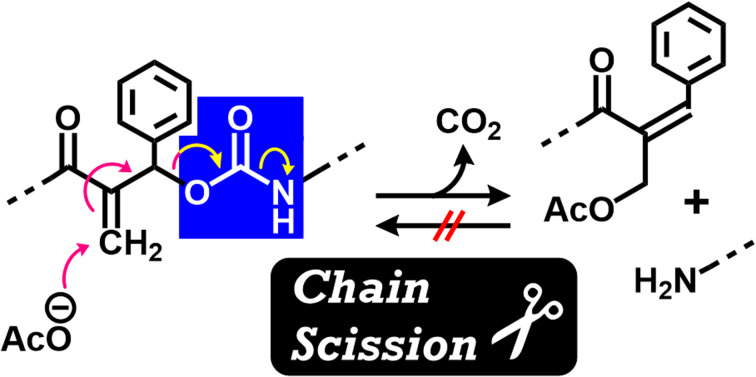

## Introduction

Poly(conjugated ester)s (PCEs) are unsaturated polyesters containing acrylate skeletons in their backbone. PCEs are thermosetting resins similar to common unsaturated polyesters.^1^ Given that an acrylic skeleton exhibits much higher reactivity than a maleic skeleton, which performs the curing-reaction site of common unsaturated polyesters, PCEs exhibit sufficient curability even with a small content of acrylic skeleton^2^ and are photocurable.^3^ Therefore, PCEs have gained attention as resins for coatings and adhesives. As the acrylate skeleton accepts further modification by thiol–ene ‘click’ chemistry, PCEs have also become recognized as a reactive precursor for functional polyesters.^4–8^

In spite of the potential applications of PCEs, their synthetic strategy remains in the early research stage. Itaconic acid, a bio-based dicarboxylic acid carrying an acryloyl group, is an attractive monomer of PCEs.^2,4^ However, the polycondensation of itaconic acid and its derivatives requires a heating process and often undergoes thermal curing due to the high reactivity of acrylates.^5^ Therefore, a polymerization reaction operable at ambient or lower temperature is desirable to access PCEs. Ring-opening polymerization of cyclic acrylate (α-exomethylene lactone) is a promising approach.^9–13^ Five-,^10^ six-^9,14^ and seven^15^-membered cyclic acrylates with slight ring-strain are highly stable, which causes difficulty in successful homo-polymerization. Moreover, copolymerization with other saturated lactones has been extensively investigated. Recently, Chen *et al.* reported a new catalyst with high activity, and the homopolymerization of γ-methylene butyrolactone was achieved.^10^ Polyaddition using Morita–Baylis–Hillman (MBH) reaction of diacrylate and aldehyde were operable at ambient temperature and directly afforded PCEs.^6,7,16^ However, the resulting degrees of polymerization were not high due to the slow rate of the MBH reaction and existence of side reactions such as dimerization.

Methacrylates carrying a leaving group, such as a halogen atom at the allylic position, undergo nucleophilic conjugated substitution reaction *via* an addition–elimination (S_N_2′) mechanism.^17,18^ Recently, we applied conjugate substitution reaction to prepare PCEs.^19–21^ The polycondensation using dicarboxylic acid, bisphenols, and dithiols was employed under 25 °C, resulting in a variety of PCEs with high molecular weight (*M*_n_ > 10^4^).^19^ Notably, the resulting PCEs exhibited degradability under ambient conditions. For example, the PCE prepared from a bisphenol underwent quantitative main-chain scission by suspending within an aqueous ammonia solution.^21^ The reaction mechanism of main-chain scission was detected as the conjugate substitution reaction of ammonia and allyl-substituted methacrylate moieties in the backbone. The results suggested that some PCEs containing leaving groups, such as an acyloxy group (ester bond) at the allylic position of methacrylate moieties, are attractive materials as chemo-degradable plastics.^9,22^

**Scheme 1:**
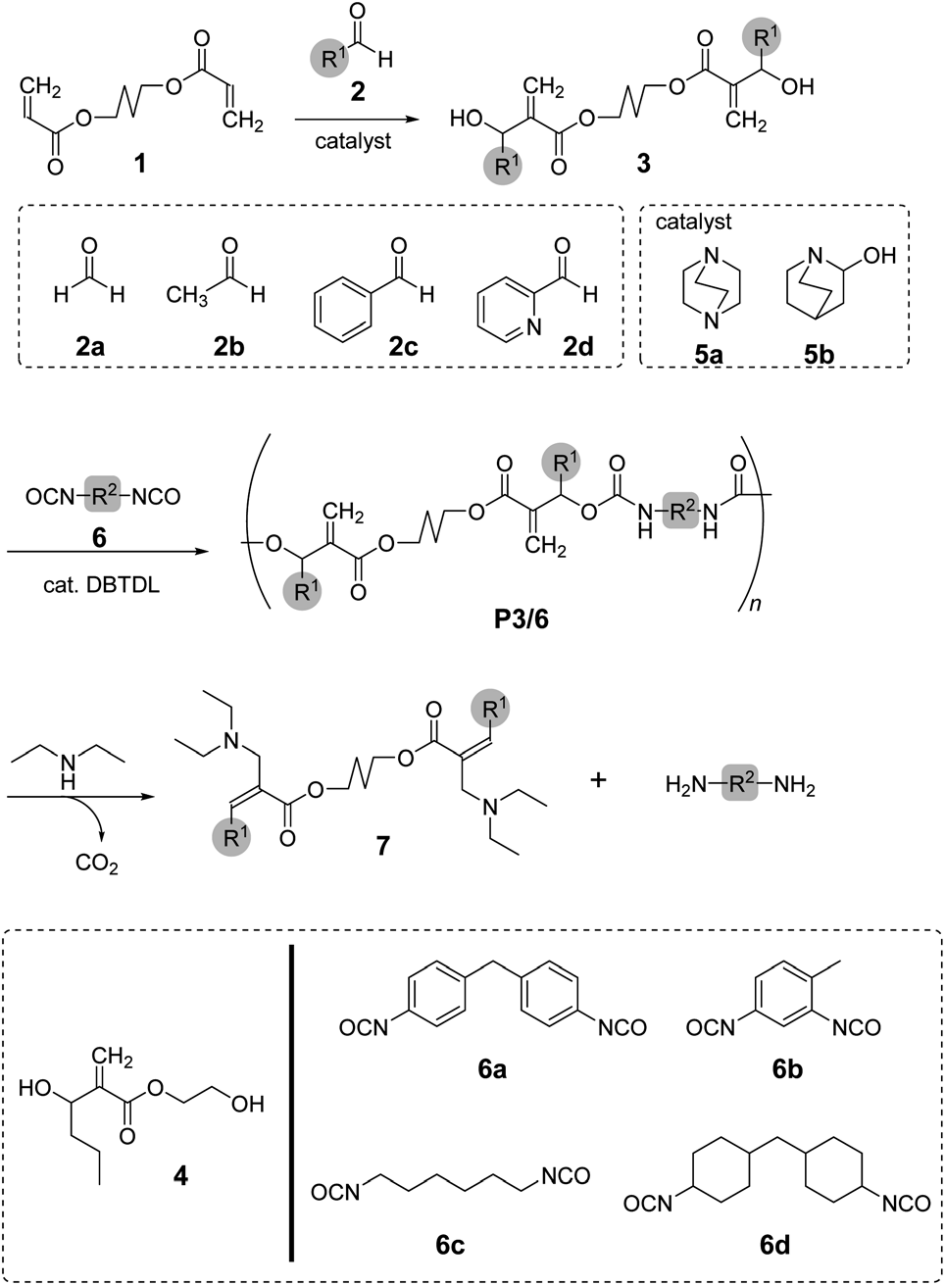
Synthesis and degradation of poly(conjugated ester–urethane)s. The substituents, R^1^ and R^2^, are corresponding to the substituents of aldehydes 2a–d and isocyanates 6a–d, respectively.

In this study, we focused on the synthesis of poly(conjugated ester–urethane)s as chemo-degradable polymers *via* conjugate substitution reactions (Scheme 1). The urethane bond is expected to be the leaving group for main-chain scission in a similar manner to the ester bond. The polyaddition of diol and diisocyanates were operable under ambient temperature, which was convenient for the synthesis of PCEs. For this polyaddition, dimethacrylate monomer 3, carrying two hydroxy groups at each allylic position, was required. Thus, we started our research with the synthesis of 3 in high yield and efficiency.

## Results and discussion

### Synthesis of dimethacrylate monomer

To synthesize poly(conjugated ester–urethane)s capable of main-chain scission by conjugate substitution reaction, the urethane bond was placed at the allylic position of the methacryloyl group (Scheme 1). Peng and Joy synthesized diol monomer 4 containing a methacrylate skeleton with hydroxy groups at allylic positions by the MBH reaction of 2-hydroxyethyl acrylate and aldehydes.^8^ The monomer afforded poly(conjugated ester–urethane)s *via* polyaddition with diisocyanate. However, the asymmetric structure of 4 was not preferable to yield several decomposed products upon main-chain scission of the polymer. Therefore, we selected symmetric diol monomers (3). The problem in the monomer synthesis was low efficiency of the MBH reaction due to a slow rate and side reactions; therefore, Peng and Joy used a large excess of acrylate and tertiary amine catalysts relative to the aldehydes.^8^ However, this technical strategy was not suitable for 3, which was generated through two MBH reactions of diacrylate.

**Scheme 2:**
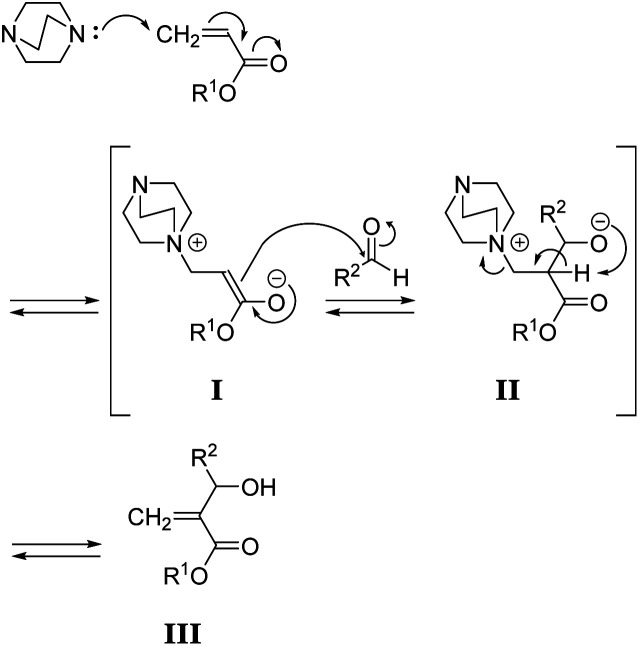
Mechanism of the MBH reaction.

The mechanism of the MBH reaction is displayed in Scheme 2. The tertiary amine is a catalyst, and a small quantity was sufficient to proceed with the reaction. However, the use of excess tertiary amine was more effective in promoting the reaction, as the rate-determining step was the E1cB reaction from intermediate II. Thus, the high concentration of amine catalyst resulted in a high concentration of II, promoting a fast reaction. Notably, Zhuang reported the efficient MBH reaction of diacrylate and formaldehyde in the presence of equimolar 5a.^23^ The protocol was also effective for 1,4-butylene diacrylate (1), resulting in 3a in a high yield (Table 1, entry 1).

**Table 1:** Synthesis of 3 by MBH reaction in bulk

Entry	Aldehyde^a^ (equimol.)	Catalyst^a^ (mol%)	Time [h]	Yield [%]
1^b^	2a (2.4)	5a (200)	23	79
2	2b (20)	5a (13)	23	85
3	2b (4.0)	5a (20)	15 days	36^c^
4	2c (5.0)	5b (47)	30	40
5	2d (2.2)	5b (40)	30 min	44

a Feeding ratio to 1.

b In H_2_O (50 mL) and CH_3_CN (80 mL) solutions of 1 (101 mmol).

c Estimated from gas chromatography.

In contrast, a high concentration of aldehyde increased the concentration of II. This approach was effective for liquid aldehyde that could be used as both a reagent and solvent. The MBH reaction of 1 and excess acetaldehyde (2b) in bulk for 24 h resulted in 3b in a high yield (entry 2). Notably, the same reaction in diluted condition afforded 3b in 36% yield even after 15 days (entry 3), suggesting the acceleration of the MBH reaction in bulk.

3-Quinuclidinol (5b) was reported as an excellent catalyst for the MBH reaction between methyl acrylate and benzaldehyde (2c).^24,25^ Thus, the MBH reactions of 1 and 2c were conducted under a similar condition (entry 4), resulting in 3c in moderate yield. 2-Pyridinecarboxyaldehyde (2d) was reported as an excellent reagent for the MBH reaction.^26,27^ The MBH reaction of 1 with nearly equimolar 2d was investigated (entry 5). After 30 min, the complete consumption of 1 was detected by gas chromatography, and 3d was isolated in a moderate yield (44%) after purification by column chromatography.

### Synthesis of poly(conjugated ester–urethane)s

**Fig. 1:**
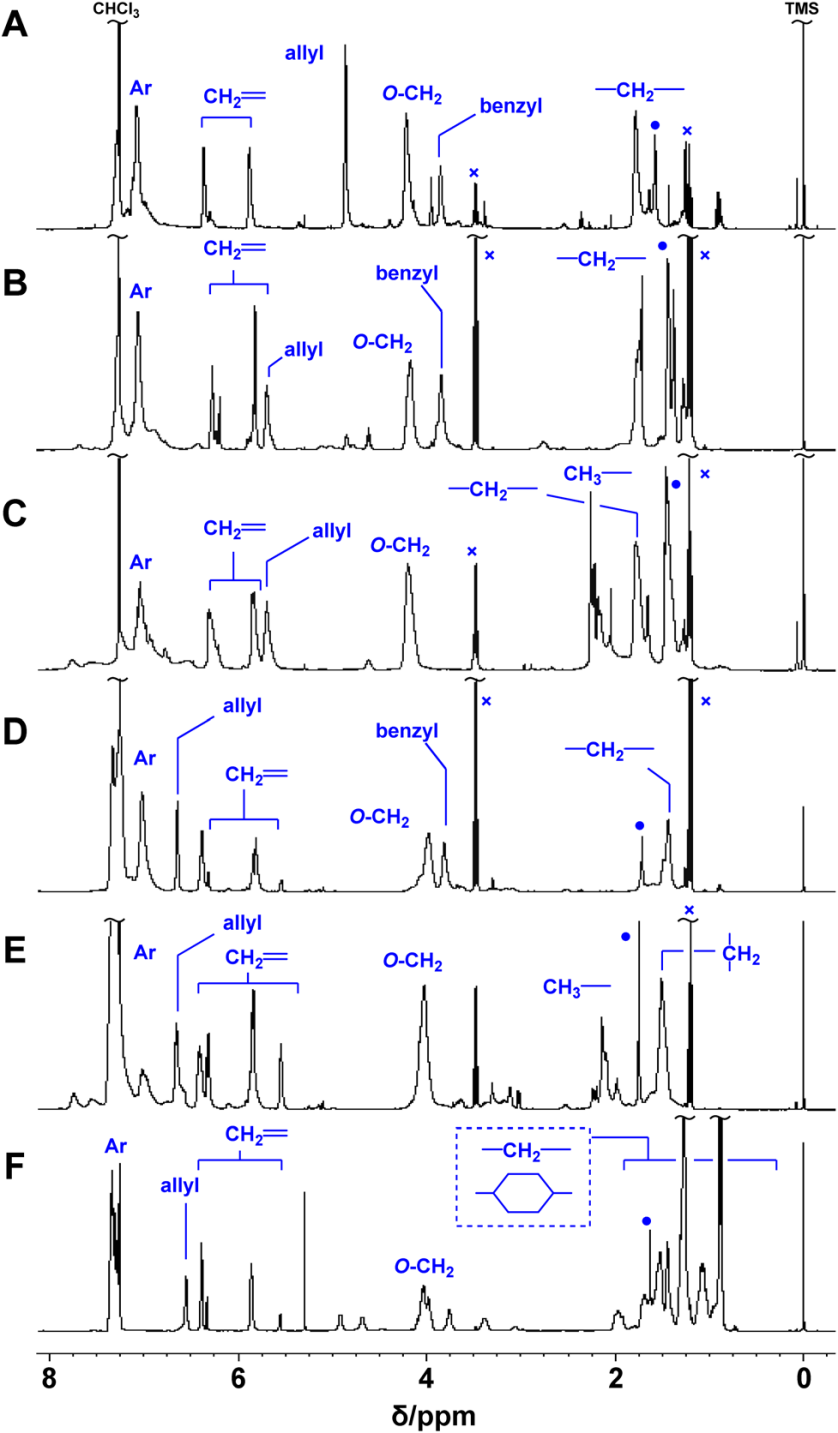
^1^H NMR spectra of P3a/6a (A), P3b/6a (B), P3b/6b (C), P3c/6a (D), P3c/6b (E), and P3c/6d (F) (400 MHz, CDCl_3_, 298 K). ●: H_2_O, ×: Et_2_O.

Poly(conjugated ester–urethane)s were synthesized by the polyaddition of 3 and various diisocyanates (6) in the presence of di-*n*-butyltin dilaurate (DBTDL) (Scheme 1 and Table 2). The polyadditions of 3a and 6a afforded polyurethane P3a/6a (Table 2, entry 1). Fig. 1A shows the ^1^H NMR spectrum of the resulting polymer, P3a/6a. No signals assignable to the chain end were observed. Thus, the *M*_n_ lower than 10^4^ can be attributed to the cyclization of the relatively smaller hydrodynamic radius than the standard polymer, poly(methyl methacrylate)s, owing to the self-aggregation by hydrogen bonding of urethane bonds.

**Table 2:** Polyadditions of 3 and 6 to poly(conjugated ester–urethane)s

Entry^a^	3	6	Yield [%]	*M*_n_ b	*Đ* b	*T*_d10_ c [°C]
1	3a	6a	92	5800	1.58	
2	3b	6a	56	4000	1.68	155
3	3b	6b	77	8900	2.19	155
4	3c	6a	96	4500	1.60	197
5	3c	6b	41	2400	1.21	239
6^d^	3c	6c	Gelation			
7^d^	3c	6d	80	4500	1.55	
8	3d	6a	— (Side reactions)			

a Equimolar monomers were reacted in CH_2_Cl_2_ at room temperature in the presence of DBTDL.

b Determined from SEC (0.01 M LiBr/DMF, 40 °C, PMMA standards).

c 10% weight loss temperature under N_2_ atmosphere.

d Polymerized in CHCl_3_ at 50 °C.

Polymerization with other monomers afforded similar results, except for entries 6 and 8. Thus, the primary alcohol 3a and secondary alcohols 3b and 3c have similar reactivity to isocyanate, at least under these conditions. For entry 6, polyaddition of 3c and 6c was conducted at 50 °C but gelation was observed during the reaction. Given that the polymerization with 6d afforded a soluble product, the gelation was not attributed to the radical polymerization of methacrylate moieties. In fact, a methacrylate bearing bulky substituents at the allylic position was inactive in radical polymerization.^28^ Polyaddition with 3d was attempted; however, intense heat generation was observed and resulted in no yield of the polymeric product. The pyridyl group of 3d could react with isocyanate and catalyze the conjugate substitution reaction of the resulting urethane unit leading to main-chain scission. Thus, 3d was not a suitable monomer for PCEs, although the synthesis was achieved within a short reaction time (Table 1, entry 5). The thermal properties of the obtained polymers were evaluated using thermogravimetry-differential thermal analysis (TG-DTA). Joy *et al.* have reported that the polyurethane of 4 was not thermally stable and decomposed below 200 °C.^8^ Similar tendency was observed for polymers of 3 and 6 (Table 2).

**Scheme 3:**
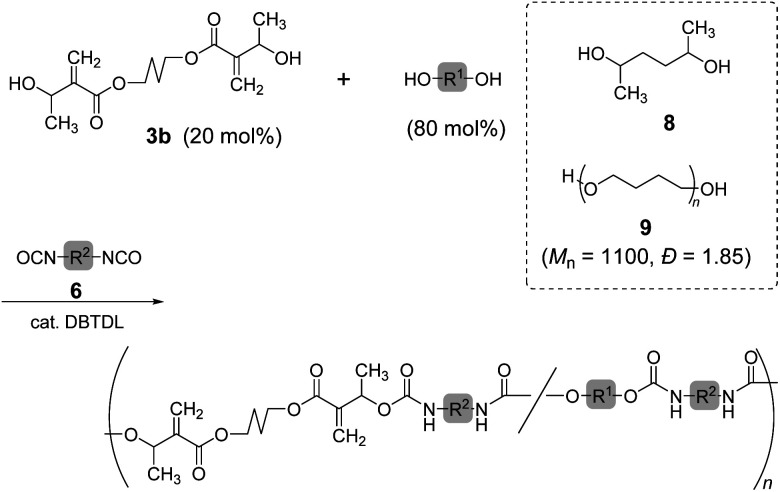
Copolymerization of 3b and diols with 6.

Copolymerization of 3b and 6b with 2,5-hexanediol (8) was also investigated (Scheme 3; Table 3, entry 1). The ^1^H NMR spectrum of the resulting polymer suggested reasonable composition ([3b]/[8] = 0.22/0.78) corresponding to the feeding ratio ([3b]/[8] = 0.20/0.80). Moreover, a polyurethane elastomer was prepared by polyaddition of 3b, 6a, and poly(tetrahydrofuran) (9) (*M*_n_ = 1100, *Đ* = 1.85). A mixture of 3b (20 mol%) and 9 (80 mol%) was reacted with 6a in *N*,*N*-dimethylformamide (DMF) under ambient temperature for 48 h. However, the resulting polyurethane had lower *M*_n_ than expected (entry 2, *M*_n_ = 5000, *Đ* = 1.91). The ^1^H NMR spectrum of the polyurethane indicated the complete consumption of secondary alcohol in the chain end. Thus, the secondary alcohol moieties of 3b were converted to urethane bonds. Consequently, the low *M*_n_ was attributed to the cyclization or inefficient reaction of the isocyanate-chain end and 6a. In order to improve the low molecular weight, polyaddition was performed with the prepolymer method. That is, the oligomerization of 6a and 9 (80 mol%) was operated for 24 h, and 3b (20 mol%) was subsequently added. After another 24 h, the reaction mixture changed to a highly viscous liquid. Finally, the polyurethane with a higher molecular weight (entry 3, *M*_n_ = 14 000, *Đ* = 2.22) was obtained.

**Table 3:** Copolymerization of 3b and diols with 6 to form polyurethanes

Entry^a^	Diol	6	Yield [%]	Comp. [%]		*M*_n_ b	*Đ* b
				3b	Diol		
1	8	6b	77	22	78	8900	2.19
2	9	6a	44	24	76	5000	1.91
3^c^	9	6a	45	21	79	14 000	2.22

a A mixture of 3b (20 mol%) and diol (80 mol%) was reacted with equimolar 6 in DMF at room temperature in the presence of DBTDL.

b Determined from SEC (0.01 M LiBr/DMF, 40 °C, PMMA standards).

c Prepolymer method.

### Chain scission of poly(conjugated ester–urethane)s

**Fig. 2:**
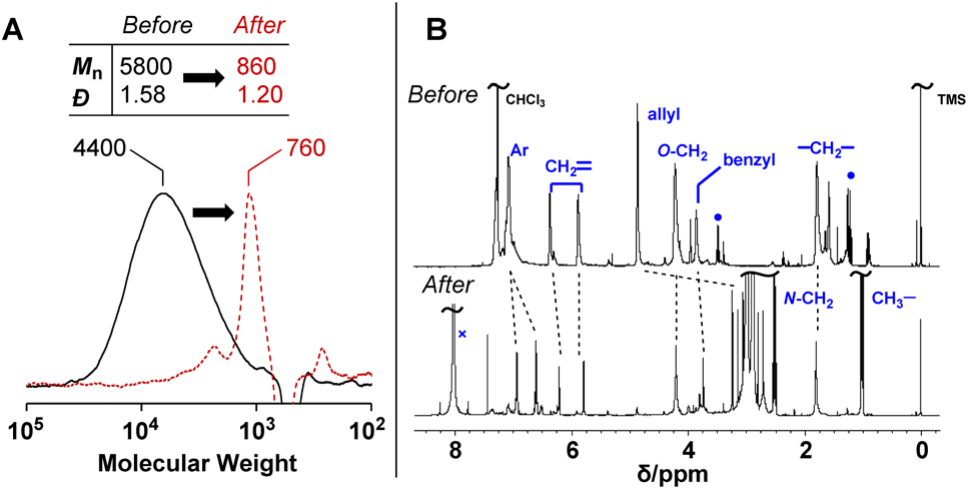
SEC curves (A) and ^1^H NMR spectra (B) before and after the degradation of P3a/6a using Et_2_NH. ●: Et_2_O, ×: DMF.

**Scheme 4:**
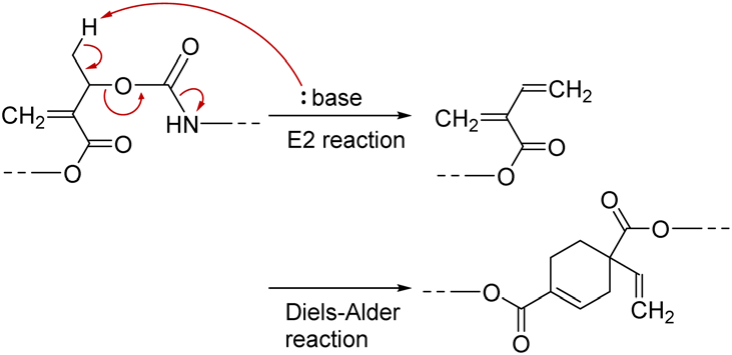
Proposed side reaction in main-chain scission of P3b/6a using Et_2_NH.

PCEs carrying a leaving group at the allylic position undergo main-chain scission by nucleophilic conjugate substitution reaction.^19,21^ Moreover, the obtained poly(conjugated ester–urethane)s had carbamate moieties (urethane bond at the allylic position) and were expected to be chemo-degradable. Thus, P3a/6a in DMF was treated with diethylamine (Et_2_NH) equimolar to the methacrylate moieties (Scheme 1). Fig. 2A shows the SEC curves before and after the reaction. Notably, the molecular weight decreased significantly after the reaction. The peak-top molecular weight (*M*_p_) changed from 4400 to 760. The ^1^H NMR spectra (Fig. 2B) suggested the quantitative conjugate substitution reaction. Similar quantitative chain scission was observed for P3c/6a and P3c/6d (Fig. S10 and S11†). In contrast, the chain scission of P3b/6a using an equimolar Et_2_NH for the methacrylate moieties resulted in the oligomeric products (*M*_p_ = 1400), while the ^1^H NMR spectrum after the reaction suggested that the degree of reaction was 65% (Fig. S12†). Comparing the results of poly(conjugated ester–urethane)s, the methyl pendant at the allylic position of P3b/6a seems to prevent chain scission; however, a possible side reaction is the elimination reaction at the methyl substituent in the E2 mechanism and the subsequent Diels–Alder reaction (Scheme 4),^29,30^ although the details were unclear in the current case.

**Scheme 5:**
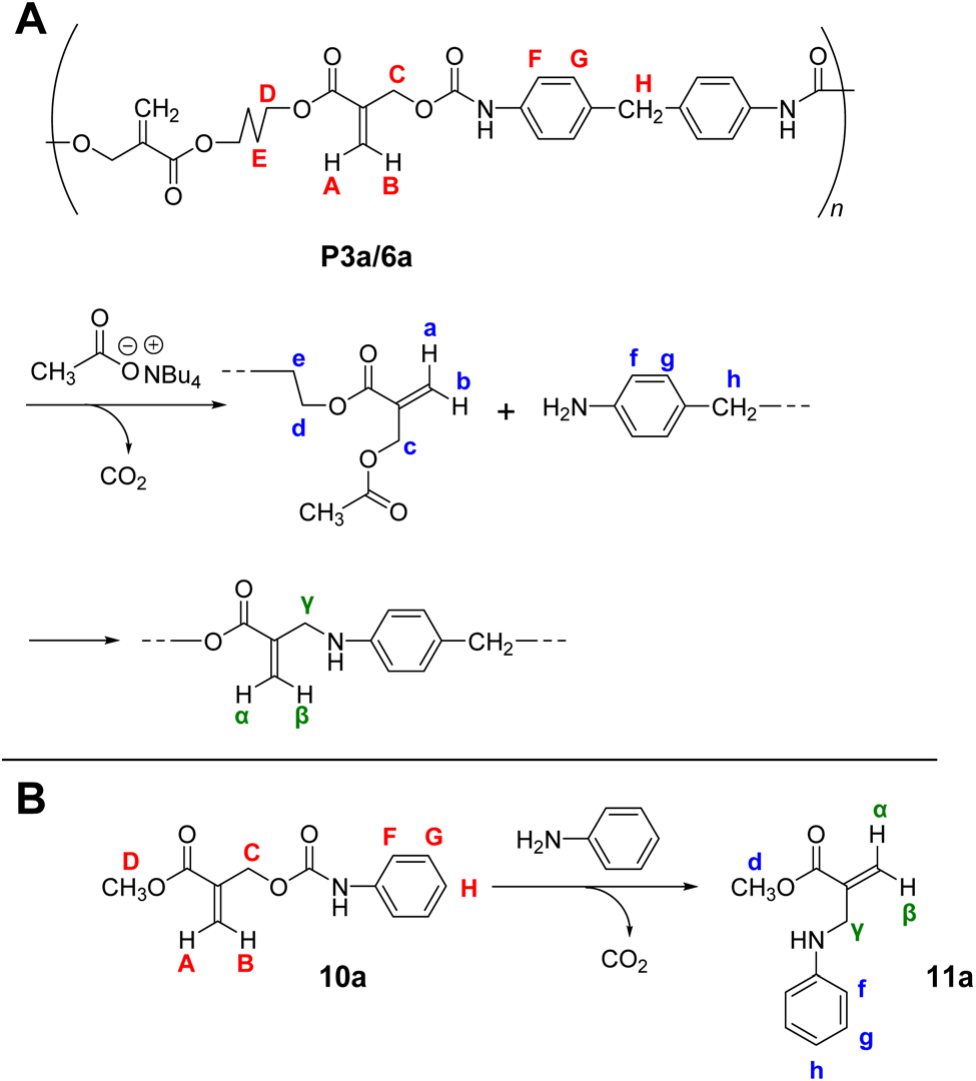
Main-chain scission of P3a/6a using Bu_4_N^+^·^−^OAc (A) and its model reaction (B). Labels correspond to Fig. 3.

**Fig. 3:**
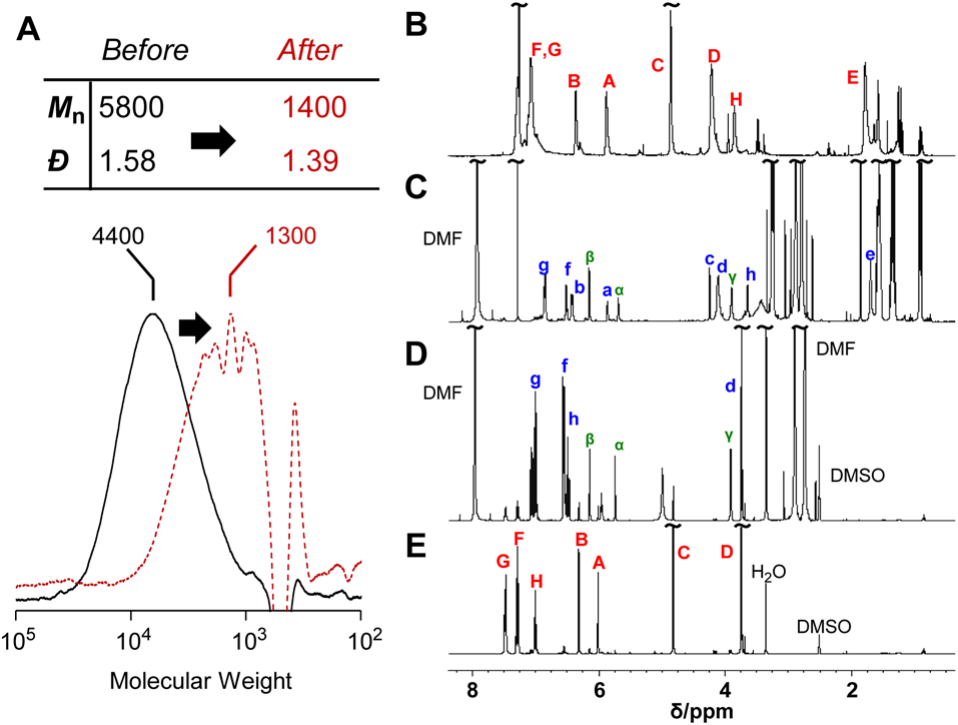
SEC curves (A) and ^1^H NMR spectra (400 MHz, CDCl_3_, 298 K) before (B) and after (C) the main-chain scission of P3a/6a using Bu_4_N^+^·^−^OAc. ^1^H NMR spectra (400 MHz, DMSO-*d*_6_, 298 K) after (D) and before (E) the model reaction of 10a. Labels for assignments correspond to Scheme 5.

The reversibility of the conjugate substitution reaction depends on the acidity of the liberating component and the nucleophile.^18^ Therefore, the treatment of PCEs containing an acyloxy group at the allylic position with carboxylate anions should result in reversible and incomplete chain scission. Nevertheless, the chain scission of poly(conjugated ester–urethane)s was expectedly irreversible, as the conjugate substitution reaction follows a decarboxylation process. The chain scission of P3a/6a by tetrabutylammonium acetate (Bu_4_N^+^·^−^OAc) in DMF was examined (Scheme 5A). The SEC curves before and after the reaction (Fig. 3A) suggest chain scission, although the remaining oligomeric products implied an incomplete reaction. In contrast, the ^1^H NMR spectra of the product (Fig. 3C) indicated the disappearance of the signals of virgin polymer, A–C (Fig. 3B), suggesting the occurrence of a quantitative conjugate substitution reaction. To explain the contradiction of incomplete chain scission and complete conjugate substitution, a side reaction of re-attack of the decomposed chain end was postulated (Scheme 5A). Moreover, the conjugate substitution reaction with carboxylate anion afforded CO_2_ and an aniline-chain end, and the latter attacked the inner unreacted methacrylate moieties or the terminal-reacted methacrylate moiety. To examine the hypothesis, a model reaction using methacrylate 10a and aniline was employed (Scheme 5B). The comparison of ^1^H NMR spectra before and after the reaction (Fig. 3D and E) revealed the characteristic signals assignable to the substituted product 11a. Then, the ^1^H NMR spectrum of the decomposed polymer (Fig. 3C) was confirmed again, and signals α–γ, indicating the aniline-substituted moieties, were clearly observed. Thus, the chain scission of P3a/6a by carboxylate anion was irreversible but underwent recombination.

**Scheme 6:**
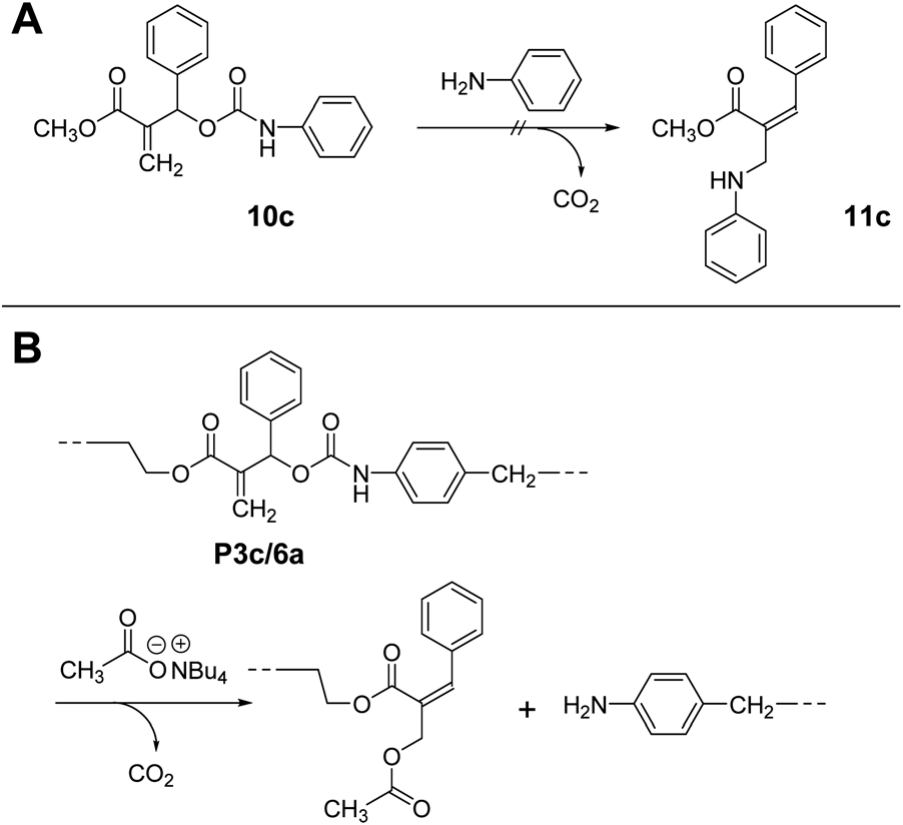
A model reaction of chain scission of 10c by aniline (A). Quantitative chain scission of P3c/6a using Bu_4_N^+^·^−^OAc (B).

Notably, 10c, a methacrylate bearing a phenyl substituent, did not undergo conjugate substitution with aniline (Scheme 6A). The product, 11c, was more stable than 10c due to the expanded conjugated system. Therefore, this reaction should proceed irreversibly, although the result was that no reaction occurred. Consequently, this reaction was under kinetic control due to the large steric hindrance of the phenyl substituent. Then, the chain scission of P3c/6a was examined using Bu_4_N^+^·^−^OAc (Scheme 6B). As expected, the chain scission proceeded quantitatively, confirmed by SEC and ^1^H NMR spectra (Fig. S14†). Consequently, the allyl substituent was the key to achieving main-chain scission using a weak nucleophile, the carboxylate anion.

## Conclusions

Diol monomers were derived in one step from diacrylates *via* the MBH reaction. MBH reactions are complex and involve side reactions; however, the target diol was obtained in a relatively high yield under the conditions established from understanding the reaction mechanism. A series of poly(conjugated ester–urethane)s were prepared by polyaddition, which underwent a quantitative main-chain scission through a conjugate substitution reaction with a secondary amine. Although the reaction with carboxylate anions was irreversible due to decarboxylation, a side reaction of recombination by the liberated aniline-chain end occurred. This side reaction was kinetically suppressed by the substitution of a phenyl group at the allylic position. Moreover, the introduction of allyl substituents was an important factor in enhancing the degradability of the polymer. In designing degradable polymers, the focus is usually on the backbone structure, particularly on the selection of the bonds that serve as the degradation points. This study shows that the design of the pendant group is also important from the viewpoint of kinetic reaction control.

## Author contributions

T. N. wrote the manuscript, re-analyzed the measurement results, and formatted the paper. A. T. employed all experiments. Y. A. technically supported A. T.’s first several experiments. Y. K. designed the molecules, synthetic routes, and supervised this research.

## Conflicts of interest

There are no conflicts to declare.

## Supplementary Material

RA-013-D3RA03461E-s001
