# Supplementary material for: Unsaturated polyurethanes degradable by conjugate substitution reactions with amines and carboxylate anions
Source: RSC Adv. 2023 Jul 7;13(29):20336–41. doi: 10.1039/d3ra03461e (PMC10327625; doi:10.1039/d3ra03461e)
Supplement: RA-013-D3RA03461E-s001 [file RA-013-D3RA03461E-s001.pdf]

Electronic Supplementary Material (ESI) for RSC Advances.
This journal is © The Royal Society of Chemistry 2023

Supporting Information

Unsaturated polyurethanes degradable by conjugate substitution reactions with amines and carboxylate anions

Takumi Noda,[a] Anri Tanaka,[a] Yosuke Akae [a,b] and Yasuhiro Kohsaka [a,c]

Experimental

Materials

1,4-Butylene diacrylate (**1**), and methyl α-(hydroxymethyl)acrylate were kind gifts from Osaka Organic Chemical Industry Ltd. Formaldehyde aqueous solution (**2a**, 37wt%), acetaldehyde (**2b**), benzaldehyde (**2c**), 2-pyridylaldehyde (**2d**), chloroform, *N*,*N*-dimethylformamide, and tin(II) 2-ethylhexanoate were purchased from FUJIFILM Wako Pure Chemical Corporation. 1,4-Diazabicyclo[2.2.2]octane (**5a**), 3-quinuclidinol (**5b**), di-*n*-butyltin dilaurate (DBTDL), methylenediphenyl 4,4'-diisocyanate (**6a**), tolylene-2,4-diisocyanate (**6b**), hexamethylene diisocyanate (**6c**), dicyclohexylmethane 4,4'-diisocyanate (**6d**), trifluoromethanesulfonic anhydride, 2,5-hexanediol (**8**), diethylamine, tetrabutylammonium acetate, phenyl isocyanate, methyl acrylate, and aniline were purchased from Tokyo Kasei Kogyo Co., Ltd. Chloroform-d_1 ($CDCl_3$, 99.8 atom % D with 0.03vol% tetramethylsilane), dimethyl sulfoxide-d_6 (DMSO-d_6, 99.9 atom % D), dehydrated dichloromethane, and dehydrated tetrahydrofuran were purchased from Kanto Chemical Co., Inc. Acetonitrile, acetone, hexane, ethyl acetate, diethyl ether, and methanol were purchased from Yoneyama Yakuhin Kogyo Co., Ltd. 1,4-Dioxane was purchased from Kishida Chemical Co., Ltd.

Instruments

^{1}H and ^{13}C NMR spectra were recorded in $CDCl_3$ or DMSO-d_6 on an AVANCE NEO (Bruker) spectrometers. Chemical shifts in ^{1}H and ^{13}C NMR spectra were referred to the signal of tetramethylsilane (TMS) and solvent (DMSO), respectively. Gas chromatography was employed on a GC-2014 (Shimadzu) equipped with a capillary column (SH Rtx-5), using helium as a movable phase and make up gas (line rate 30 cm s^{-1}) and detected with a flame ionization detector (FID-2014). Molecular weight and its distributions were determined at 40 °C by size-exclusion chromatography (SEC) on an EXTREMA chromatograph (JASCO) equipped with two SEC columns [PL-gel, Mixed C (300 mm × 7.5 mm), Polymer Laboratories], using 0.01 M LiBr in DMF as an eluent (flow rate = 0.8 mL min^{-1}), and calibrated against standard poly(methyl methacrylate) (PMMA) samples (TSK-gel oligomer kit, Tosoh, M_n: 6.03×10^5, 2.52×10^5, 1.42×10^5, 2.91×10^4, 8.59×10^3, 4.25×10^3, 1.46×10^3, 8.30×10^2) and detected with UV (UV-4070, JASCO) and RI (RI-4030, JASCO) detectors. IR spectra were recorded on a Cary 630 FTIR spectrometer equipped with a diamond-attenuated total reflection (ATR) accessory. Thermogravimetric/differential thermal analysis (TG/DTA) was carried out from room temperature to 500 °C at a heating rate of 10 °C min^{-1} with Rigaku Thermo plus II TG8120 under an N_2 atmosphere

[a] Faculty of Textile Science and Technology, Shinshu University, 3-15-1 Tokida, Ueda, Nagano 386-8657, Japan
[b] Japan Society for the Promotion of Science (JSPS), Tokyo, Japan
[c] Research Initiative for Supra-Materials (RISM), Shinshu University, Japan
email: kohsaka@shinshu-u.ac.jp; tel: +81-268-21-5488

Synthesis

1,4-Butylene bis[*α*-(hydroxymethyl)acrylate] (**3a**)

A solution of **1** (19.8 g, 100 mmol) in **2a** (48.6 g, 600 mmol) was added dropwise to a solution of **5a** (33.6 g, 300 mmol) in acetonitrile (150 mL)-water (150 mL). The reaction mixture was stirred at 45 °C for 24 h. The product was extracted with ethyl acetate (200 mL × 2) and the combined organic layer was concentrated. The residue was purified on silica gel column chromatography (eluent:hexane / EtOAc = 1 / 3) to give **3a**. Yield, 1.29 g (5.0%). ^{1}H NMR (400 MHz, $CDCl_3$, 25 °C): δ/ppm 1.80–1.83 (m, 4H, CH_2), 2.51 (t, 2H, J = 6.3 Hz, OH), 4.23–4.26 (m, 4H, $COOCH_2$), 4.33 (d, 4H, J = 5.9 Hz, CH_2OH), 5.86 (d, 2H, J = 1.4 Hz, CH_2=), 6.26 (d, 2H, J = 0.9 Hz, CH_2=).

1,4-Butylene bis[*α*-(1-hydroxyethyl)acrylate] (**3b**)

A solution of **1** (20.4 g, 103 mmol), **2b** (89.5 g, 2.02 mol), and **5a** (1.50 g, 13.4 mmol) in 1,4-dioxane (5.6 mL) was stirred at room temperature for 4 days. The product was concentrated then the obtained residue was purified on silica gel column chromatography (eluent:hexane / EtOAc = 5 / 3) to give **3b**. Yield, 25.4 g (85.8%). ^{1}H NMR (400 MHz, $CDCl_3$, 25 °C): δ/ppm 1.38 (d, 6H, J = 6.5 Hz, CH_3), 1.82 (dt, 4H, J_1 = 5.8 Hz, J_2 = 3.0 Hz, CH_2), 2.92 (brs, 2H, OH), 4.21–4.27 (m, 4H, $COOCH_2$), 4.62 (q, 2H, J = 6.2 Hz, CH), 5.85 (s, 2H, CH_2=), 6.21 (s, 2H, CH_2=).

1,4-Butylene bis[α-(hydroxy(phenyl)methyl)acrylate] (**3c**)

A mixture of **1** (30.0 g, 152 mmol), **2c** (76.5 mL, 758 mmol), and **5b** (9.20 g, 72.3 mmol) was stirred at room temperature for 14 h. The product was concentrated then the obtained residue was purified on silica gel column chromatography (eluent:hexane / EtOAc = 1 / 1) to give **3c**. Yield, 24.8 g (39.6%). ^{1}H NMR (400 MHz, $CDCl_3$, 25 °C): δ/ppm, 1.54–1.56 (m, 4H, CH_2), 3.00 (brs, 2H, OH), 4.04–4.10 (m, 4H, $COOCH_2$), 5.54 (s, 2H, CH), 5.85 (s, 2H, CH_2=), 6.33 (s, 2H, CH_2=), 7.24–7.37 (m, 10H, ArH). ^{13}C NMR (100 MHz, $CDCl_3$, 25 °C): δ/ppm 24.9, 28.8, 62.0, 64.6, 73.0, 125.9, 126.6, 127.7, 128.3, 141.3, 142.1, 166.3.

1,4-Butylene bis[α-(hydroxy(2-pyridinyl)methyl)acrylate] (**3d**)

A solution of **1** (5.04 g, 25.5 mmol), **2d** (8.49 g, 79.3 mmol), and **5b** (1.30 g, 10.2 mmol) in 1,4-dioxane (0.5 mL) was stirred at room temperature. After stirring for 24 h, the product was extracted with chloroform (10 mL) and the organic layer was concentrated. The residue was purified on silica gel column chromatography (eluent:hexane / acetone = 1 / 1) to give **3d**. Yield, 4.62 g (43.8%). ^{1}H NMR (400 MHz, $CDCl_3$, 25 °C): δ/ppm, 1.58–1.61 (m, 4H, CH_2), 4.10 (brs, 4H, $COOCH_2$), 4.90 (brs, 2H, OH), 5.60 (s, 2H, CH), 5.96 (s, 2H, CH_2=), 6.36 (s, 2H, CH_2=), 7.20 (dd, 2H, J_1 = 7.2 Hz, J_2 = 5.2 Hz, ArH), 7.40 (dd, 2H, J = 7.9 Hz, ArH), 7.67 (td, 2H, J_1 = 7.7 Hz, J_2 = 1.6 Hz, ArH), 8.53 (d, 2H, J = 4.8 Hz, ArH).

Poly(conjugated ester urethane) (**P3/6**)

A typical polymerization procedure is as follows. To a solution of **3a** (510 mg, 1.96 mmol) and **6a** (490 mg, 1.96 mmol) in dehydrated dichloromethane (2.0 mL) under argon atmosphere, di-*n*-butyltin dilaurate (24.0 mg, 38.0 µmol) was added. The reaction mixture was stirred at room temperature for 24 h. After the reaction, diethyl ether (45 mL) was added into the reaction mixture to precipitate polymer. The formed precipitates were collected by centrifugation then were dried under reduced pressure to give **P3a/6a**. Yield, 1.29 g (92%). Other poly(urethane ester)s, **P3b/6a**, **P3b/6b**, **P3c/6a**, and

P3c/6b were synthesized following the above methods. In the synthesis of **P3c/6d**, polymerization was performed at 50 °C. Yield, **P3b/6a** (56%), **P3b/6b** (77%), **P3c/6a** (96%), **P3c/6b** (41%), and **P3c/6d** (80%).

poly(tetrahydrofuran) (**9**)

Trifluoromethanesulfonic anhydride (1.01 mL, 6.15 mmol) was added dropwise to dehydrated tetrahydrofuran (100 mL, 1.23 mol) under nitrogen atmosphere. The reaction mixture was stirred at room temperature. After stirring for 3 min, water (2 mL) was added into the reaction mixture to stop the reaction. The obtained products were added to dry-ice methanol bath to precipitate polymer. The formed precipitates were collected by decantation then were dried under reduced pressure to give **9**. Yield, 2.60 g (7.7%). M_n = 1100, $Đ$ = 1.85.

Poly(conjugated ester urethane) (**P3b/6b/8**)

To a solution of **3b** (201 mg, 0.698 mmol), **6b** (601 mg, 3.45 mmol), and **8** (328 mg, 2.78 mmol) in dehydrated dichloromethane (3.0 mL) under argon atmosphere, di-*n*-butyltin dilaurate (catalytic amount) was added. The reaction mixture was stirred at room temperature for 48 h. After the reaction, diethyl ether (40 mL) was added into the reaction mixture to precipitate polymer. The formed precipitates were collected by centrifugation then were dried under reduced pressure to give **P3b/6b/8**. Yield, 872 mg (77%).

Polyurethane elastomer (**P3b/6a/9**) by one-pot method

To a solution of **3b** (50 mg, 0.174 mmol), **6a** (219 mg, 0.876 mmol), and **9** (765 mg, 0.695 mmol) in dehydrated *N*,*N*-dimethylformamide (2.0 mL) under argon atmosphere, di-*n*-butyltin dilaurate (catalytic amount) was added. The reaction mixture was stirred at room temperature for 48 h. After the reaction, hexane (40 mL) was added into the reaction mixture to precipitate polymer. The formed precipitates were collected by centrifugation then were dried under reduced pressure to give **P3b/6a/9**. Yield, 1.29 g (44%).

Polyurethane elastomer (**P3b/6a/9**) by prepolymer method

To a solution of **9** (765 mg, 0.695 mmol) in dehydrated *N*,*N*-dimethylformamide (1.0 mL) under argon atmosphere, **6a** (220 mg, 0.880 mmol), and di-*n*-butyltin dilaurate (catalytic amount) was added. The reaction mixture was stirred at room temperature. After stirring for 24 h, a solution of **3b** (52 mg, 0.181 mmol) and di-*n*-butyltin dilaurate (catalytic amount) in dehydrated *N*,*N*-dimethylformamide (1.0 mL) was added to the reaction mixture. The reaction mixture was stirred further 24 h. After the reaction, hexane (40 mL) was added into the reaction mixture to precipitate polymer. The formed precipitates were collected by centrifugation then were dried under reduced pressure to give **P3b/6a/9**. Yield, 1.33 g (45%).

Model compound (**10a**)

To a solution of methyl α-(hydroxymethyl)acrylate (5.02 g, 43.3 mmol) in dehydrated dichloromethane (20 mL) under argon atmosphere, a solution of phenyl isocyanate (5.15 g, 43.3 mmol) and tin(II) 2-ethylhexanoate (349 mg, 0.862 mmol) in dehydrated dichloromethane (20 mL) was added. The reaction mixture was stirred at room temperature for 18 h. The product was concentrated then was diluted with acetonitrile (10 mL). The products were washed with hexane (30 mL × 3). The acetonitrile layer was concentrated then was dried under reduced pressure to give **10a**. Yield, 6.53 g

(64.2%). ^{1}H NMR (400 MHz, DMSO-d_6, 25 °C): δ/ppm 3.73 (s, 3H, $COOCH_3$), 4.81 (s, 2H, CH_2), 6.00 (d, 1H, *J* = 1.1 Hz, CH_2=), 6.31 (d, 1H, *J* = 0.8 Hz, CH_2=), 6.99 (t, 1H, *J* = 7.4 Hz, ArH), 7.28 (t, 2H, *J* = 7.9 Hz, ArH), 7.47 (d, 2H, *J* = 7.9 Hz, ArH), 9.78 (s, 1H, NH).

Model compound (**10c**)

A mixture of methyl acrylate (25.8 g, 300 mmol), **2c** (10.6 mL, 100 mmol), **5a** (11.2 g, 100 mmol),1,4-dioxane (3.0 mL), and water (3.0 mL) was stirred at room temperature for 48 h. The product was concentrated then the obtained residue was purified on silica gel column chromatography (eluent:hexane / EtOAc = 1 / 1) to give precursor of **10c**. Yield, 8.97 g (46.2%). ^{1}H NMR (400 MHz, DMSO-d_6, 25 °C): δ/ppm 3.61 (s, 3H, $COOCH_3$), 5.47 (d, 1H, *J* = 4.6 Hz, OH), 5.75 (d, 1H, *J* = 4.6 Hz, CH), 6.01 (t, 1H, *J* = 1.6 Hz, CH_2=), 6.22 (m, 1H, CH_2=), 7.22–7.34 (m, 5H, ArH).

To a solution of synthesized precursor (2.06 g, 1.04 mmol), and phenyl isocyanate (1.27 g, 1.04 mmol) in dehydrated dichloromethane (1 mL) under argon atmosphere, tin(II) 2-ethylhexanoate (84.4 mg, 0.208 mmol) was added. The reaction mixture was stirred at room temperature for 18 h. The product was concentrated then was diluted with acetonitrile (3 mL). The products were washed with hexane (10 mL × 3). The acetonitrile layer was concentrated then was dried under reduced pressure to give **10c**. Yield, 2.57 g (78.6%). ^{1}H NMR (400 MHz, DMSO-d_6, 25 °C): δ/ppm 3.68 (s, 3H, $COOCH_3$), 5.92 (s, 1H, CH_2=), 6.38 (s, 1H, CH_2=), 6.55 (s, 1H, CH), 6.99 (t, 1H, *J* = 7.3 Hz, ArH), 7.27 (t, 2H, *J* = 7.9 Hz, ArH), 7.32–7.41 (m, 5H, ArH), 7.45 (d, 2H, *J* = 8.0 Hz, ArH), 9.88 (brs, 1H, NH).

Main chain-scission

A typical chain-scission procedure is as follows. To a solution of **P3a/6a** (100 mg, 0.392 mmol for methacrylate moieties) in *N*,*N*-dimethylformamide (0.2 mL), a solution of diethylamine (equimolar to the methacrylate moieties) in *N*,*N*-dimethylformamide (0.2 mL) was added. The reaction mixture was stirred for 24 h at room temperature. After the reaction, ^{1}H NMR and SEC measurements for obtained product were performed to confirm chain-scission reaction. Chain-scission of other polyurethanes using diethylamine and tetrabutylammonium acetate as nucleophile were performed following the above methods.

Model reaction using methacrylate and aniline

A typical model reaction procedure is as follows. To a solution of **10a** (100 mg, 0.426 mmol) in *N*,*N*-dimethylformamide (0.2 mL), a solution of aniline (equimolar to the methacrylate) in *N*,*N*-dimethylformamide (0.2 mL) was added. The reaction mixture was stirred for 24 h at room temperature. After the reaction, ^{1}H NMR measurement for obtained product was performed to confirm chain-scission reaction. Model reaction using another methacrylate (**10c)** was performed following the above methods.

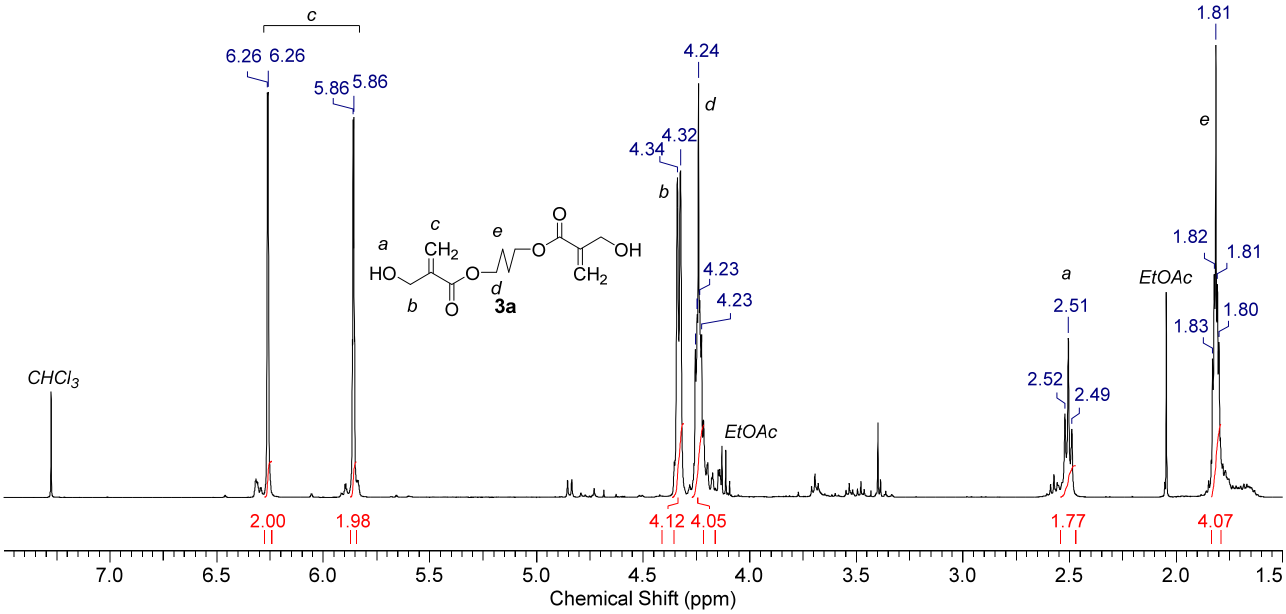

Fig. S1. ^{1}H NMR spectrum of **3a** (400 MHz, $CDCl_3$, 298 K).

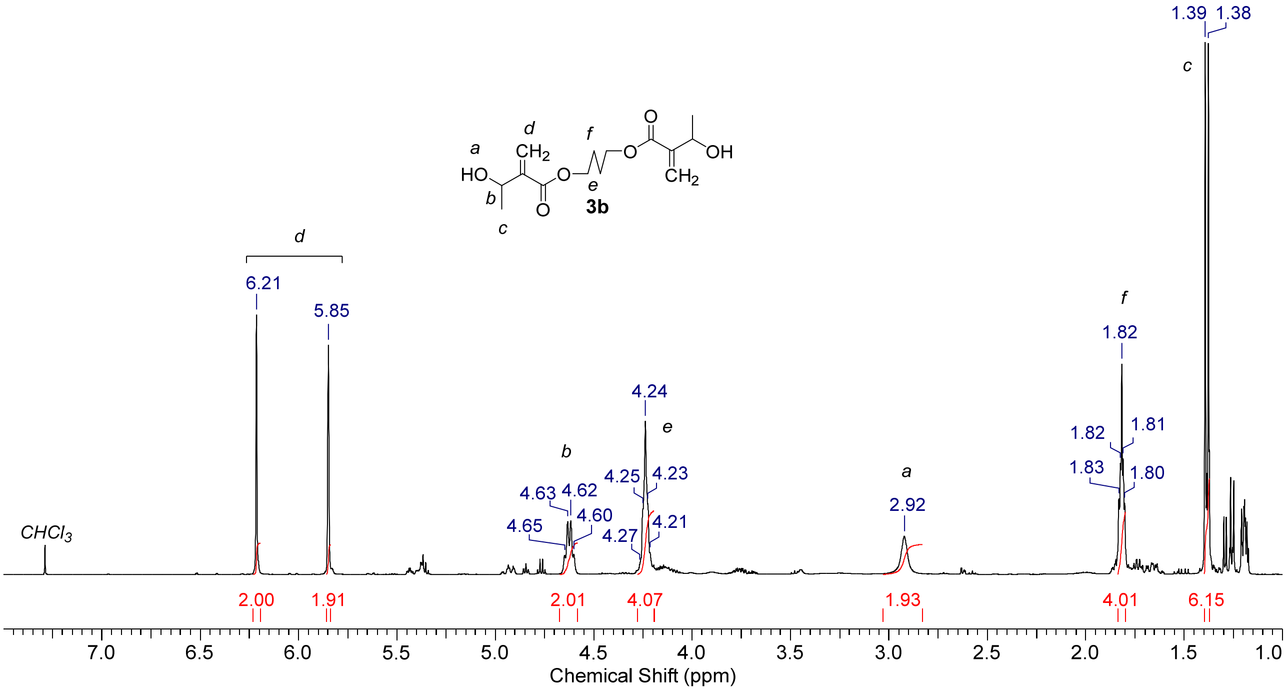

Fig. S2. ^{1}H NMR spectrum of **3b** (400 MHz, $CDCl_3$, 298 K).

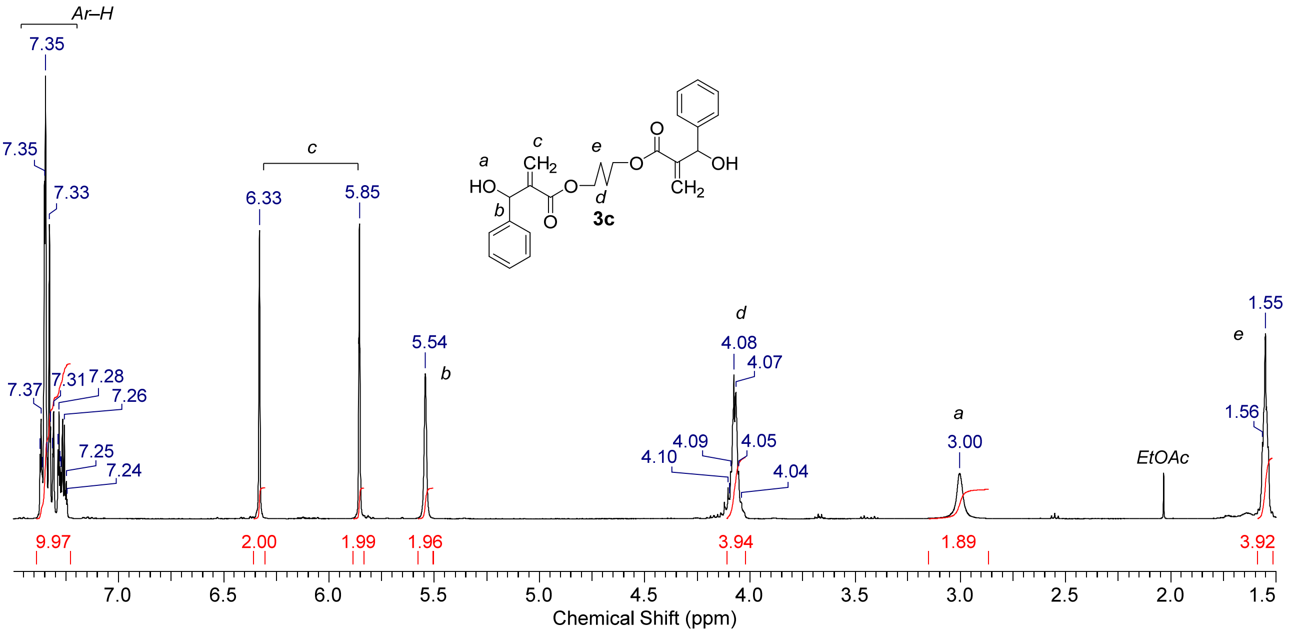

Fig. S3. ^{1}H NMR spectrum of **3c** (400 MHz, $CDCl_3$, 298 K).

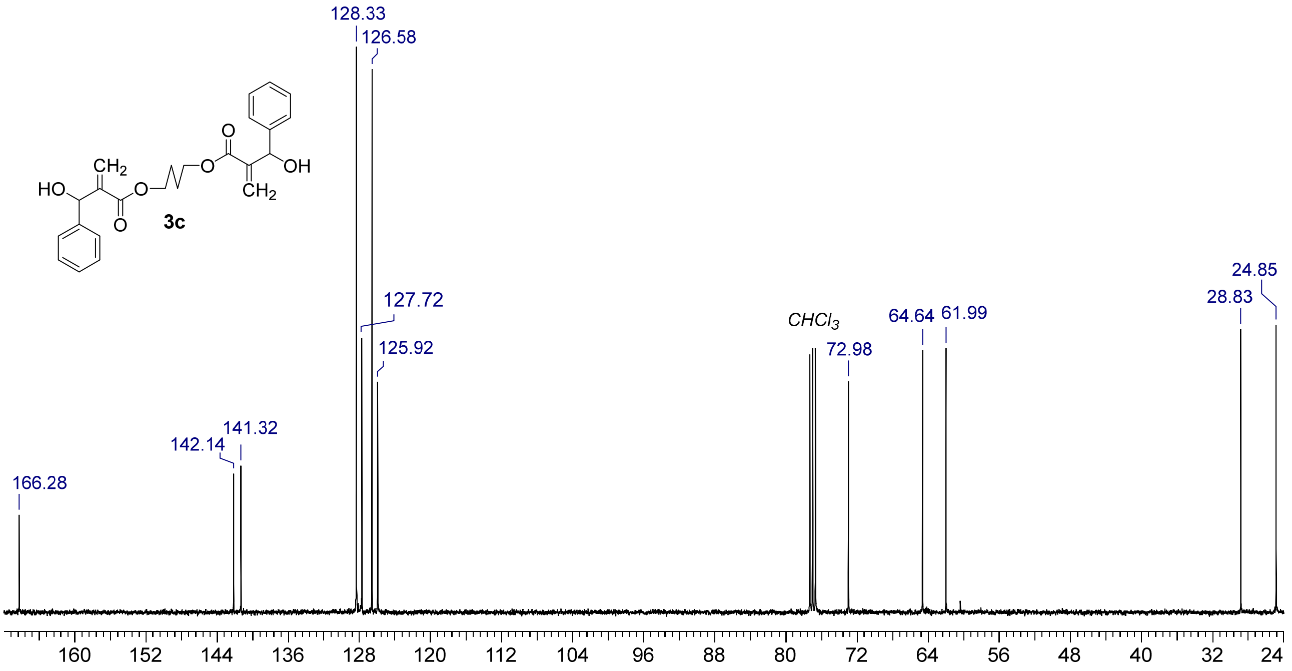

Fig. S4. ^{13}C NMR spectrum of **3c** (100 MHz, $CDCl_3$, 298 K).

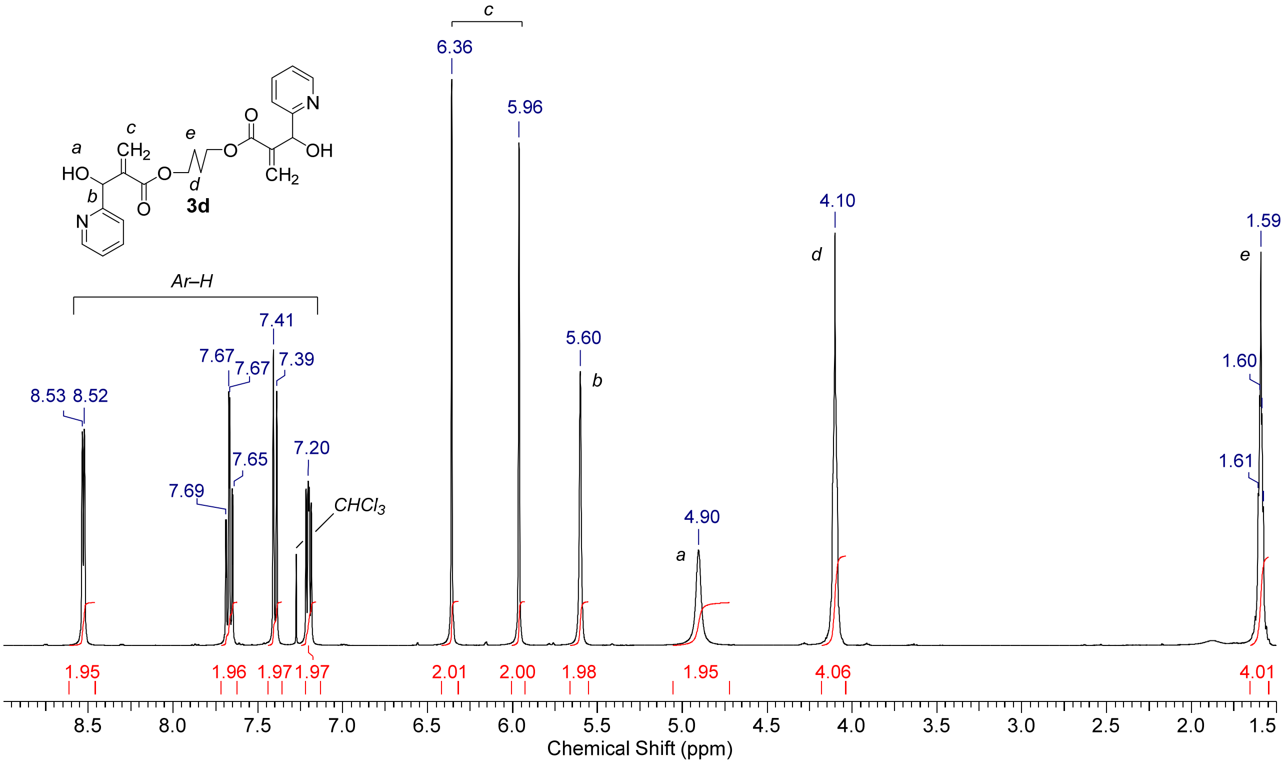

Fig. S5. ^{1}H NMR spectrum of **3d** (400 MHz, $CDCl_3$, 298 K).

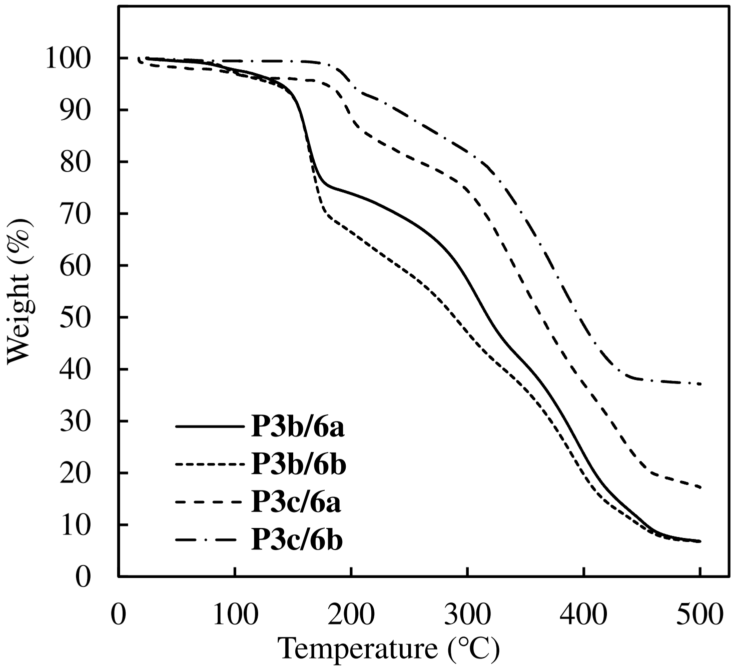

Fig. S6. TGA curves of poly(urethane ester)s.

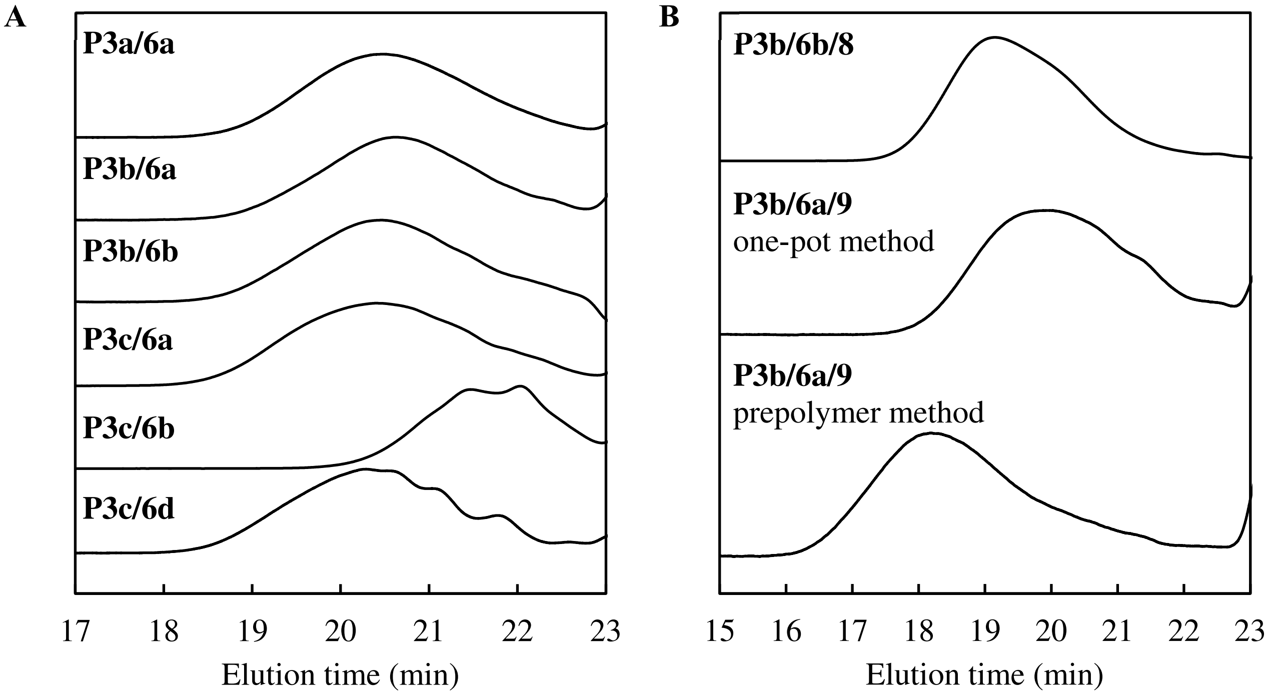

Fig. S7. SEC traces of the poly(urethane ester)s (**A**), and copoly(urethane ester)s (**B**) (0.01 M LiBr in DMF; 0.8 mL min^{-1}; 40 °C).

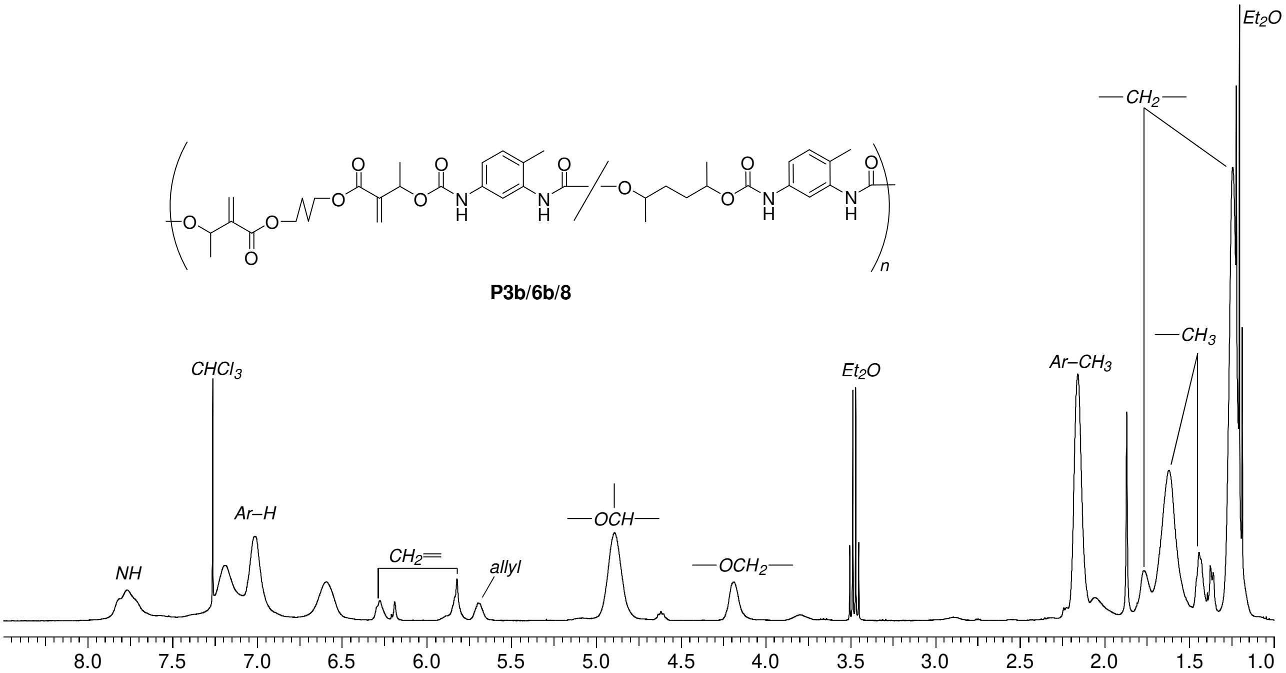

Fig. S8. ^{1}H NMR spectrum of **P3b/6b/8** (400 MHz, $CDCl_3$, 298 K).

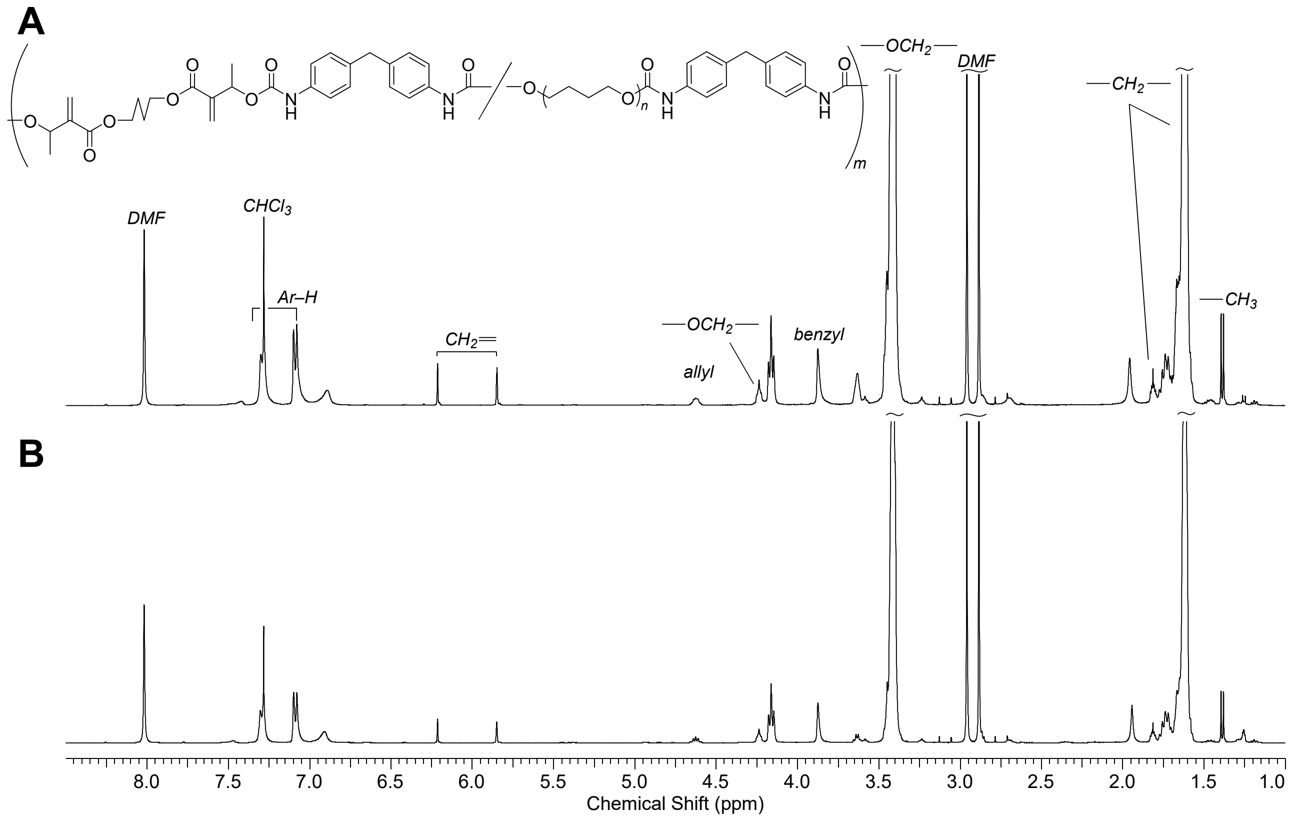

Fig. S9. ^{1}H NMR spectra of **P3b/6a/9**, synthesized by one-pot method (**A**), and synthesized by prepolymer method (**B**) (400 MHz, $CDCl_3$, 298 K).

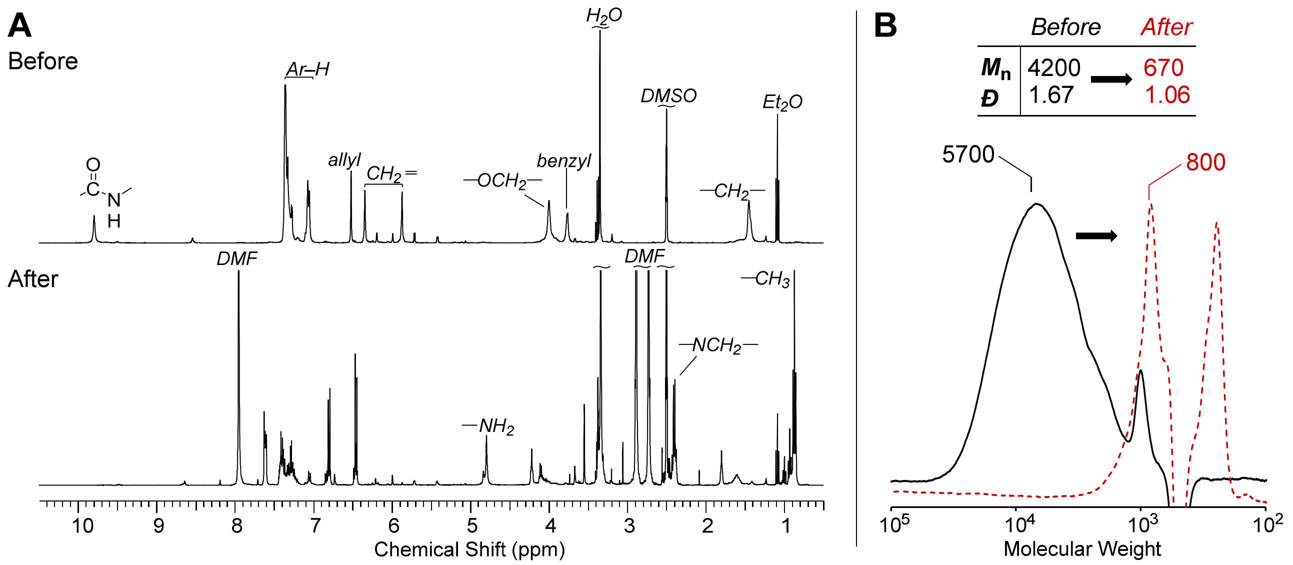

Fig. S10. ^{1}H NMR spectra (400 MHz, DMSO-d_6, 298 K) (**A**) and SEC traces (0.01 M LiBr in DMF; 0.8 mL min^{-1}; 40 °C) (**B**) before and after the degradation of **P3c/6a** using Et_2NH. **P3c/6a** was prepared in a different lot from that in Table 4, Entry 4.

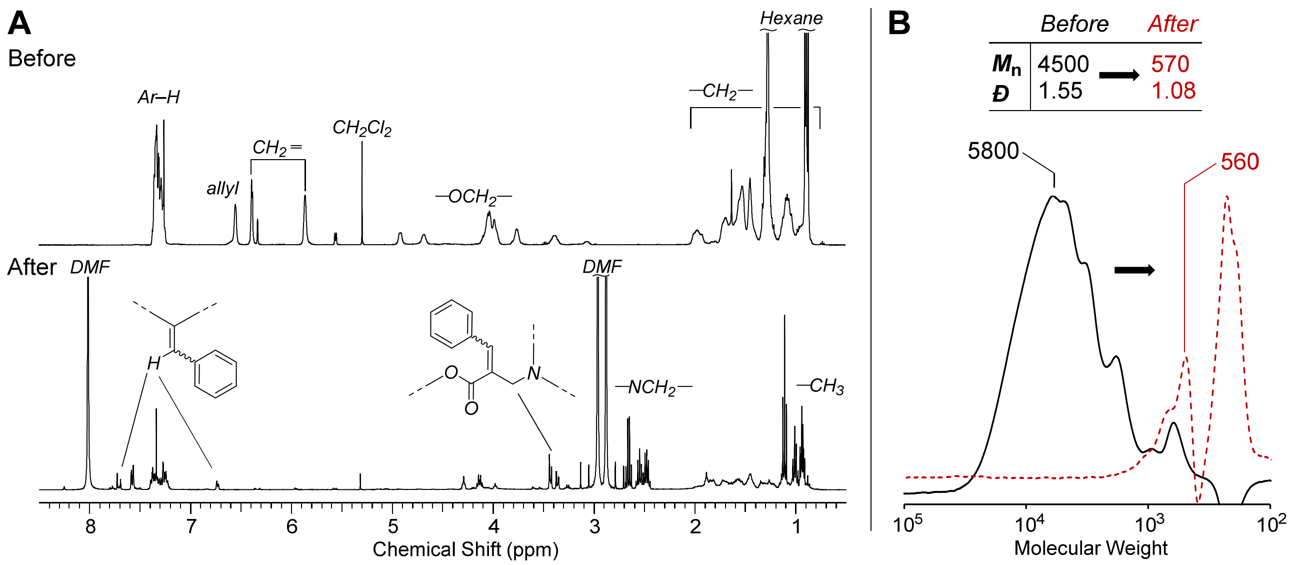

Fig. S11. ^{1}H NMR spectra (400 MHz, $CDCl_3$, 298 K) (**A**) and SEC traces (0.01 M LiBr in DMF; 0.8 mL min^{-1}; 40 °C) (**B**) before and after the degradation of **P3c/6d** using Et_2NH.

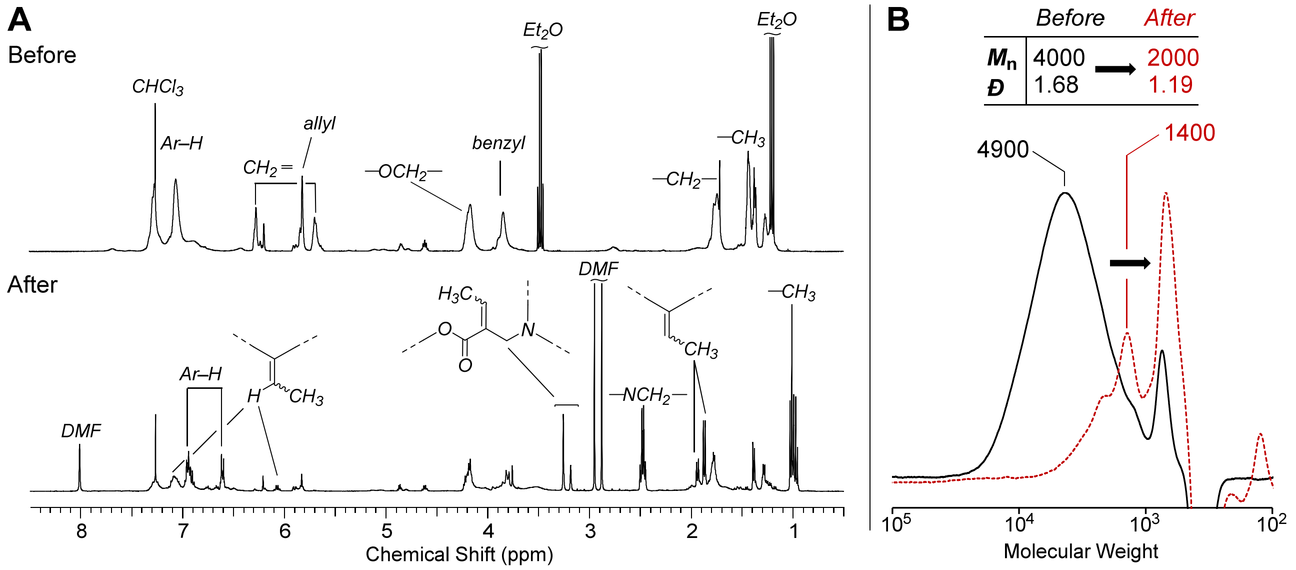

Fig. S12. ^{1}H NMR spectra (400 MHz, $CDCl_3$, 298 K) (**A**) and SEC traces (0.01 M LiBr in DMF; 0.8 mL min^{-1}; 40 °C) (**B**) before and after the degradation of **P3b/6a** using Et_2NH.

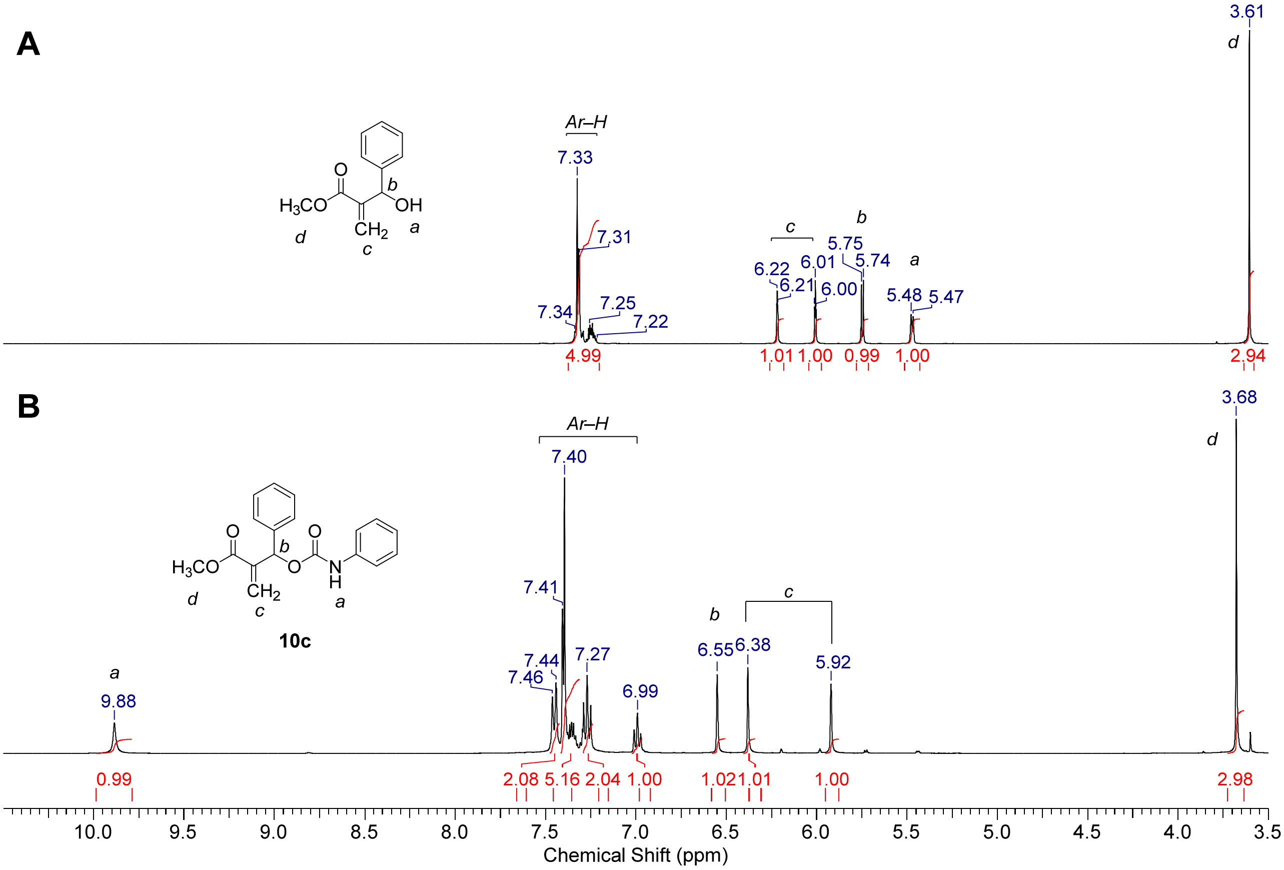

Fig. S13. ^{1}H NMR spectra of synthesized precursor (**A**), and **10c** (**B**) (400 MHz, DMSO-d_6, 298 K).

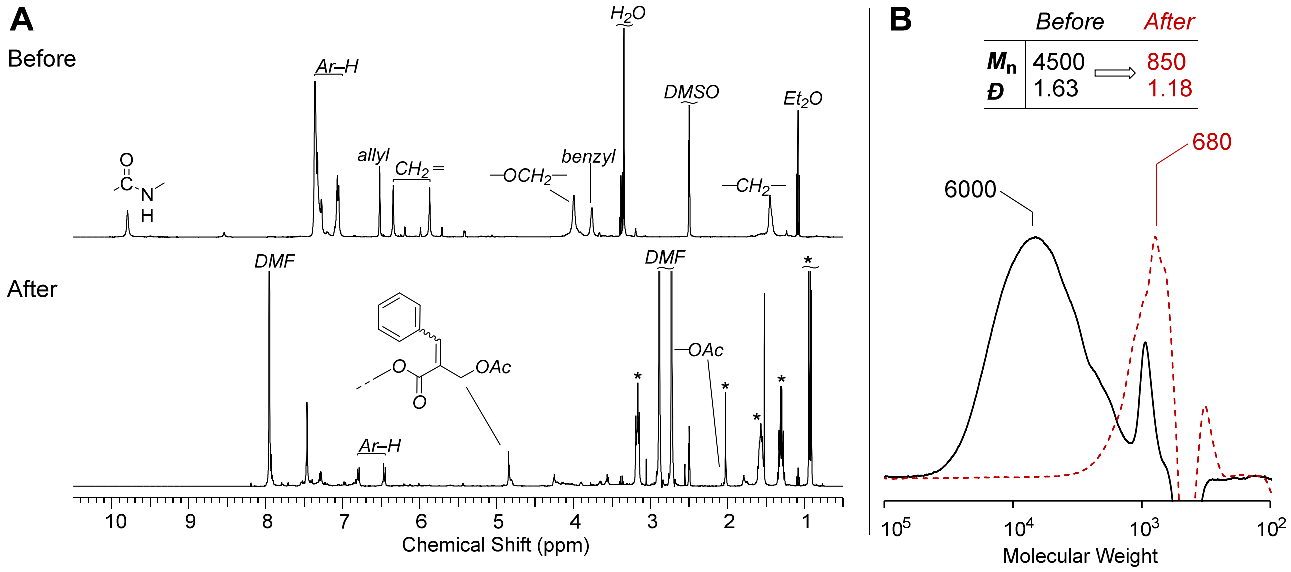

Fig. S14. ^{1}H NMR spectra (400 MHz, DMSO-d_6, 298 K. *:$Bu_4N^+ \cdot {}^-OAc$) (**A**) and SEC traces (0.01 M LiBr in DMF; 0.8 mL min^{-1}; 40 °C) (**B**) before and after the degradation of **P3c/6a** using $Bu_4N^+ \cdot {}^-OAc$. **P3c/6a** was prepared in a different lot from that in Table 4, Entry 4.
